# A Method of Multiple Dynamic Objects Identification and Localization Based on Laser and RFID

**DOI:** 10.3390/s20143948

**Published:** 2020-07-16

**Authors:** Wenpeng Fu, Ran Liu, Heng Wang, Rashid Ali, Yongping He, Zhiqiang Cao, Zhenghong Qin

**Affiliations:** 1 School of Information Engineering, Southwest University of Science and Technology, Mianyang 621010, China; wenpeng_fu628@mails.swust.edu.cn (W.F.); wh839@swust.edu.cn (H.W.); rashidcs@uot.edu.pk (R.A.); yongping_he@mails.swust.edu.cn (Y.H.); caozhiqiang@mails.swust.edu.cn (Z.C.); qinzhy527@mails.swust.edu.cn (Z.Q.); 2 Engineering Product Development, Singapore University of Technology and Design, Singapore 487372, Singapore; 3 Department of Computer Science, University of Turbat, Balochistan 92600, Pakistan

**Keywords:** dynamic objects identification and localization, laser cluster, radial velocity similarity, Pearson correlation coefficient, particle filter

## Abstract

In an indoor environment, object identification and localization are paramount for human-object interaction. Visual or laser-based sensors can achieve the identification and localization of the object based on its appearance, but these approaches are computationally expensive and not robust against the environment with obstacles. Radio Frequency Identification (RFID) has a unique tag ID to identify the object, but it cannot accurately locate it. Therefore, in this paper, the data of RFID and laser range finder are fused for the better identification and localization of multiple dynamic objects in an indoor environment. The main method is to use the laser range finder to estimate the radial velocities of objects in a certain environment, and match them with the object’s radial velocities estimated by the RFID phase. The method also uses a fixed time series as “sliding time window” to find the cluster with the highest similarity of each RFID tag in each window. Moreover, the Pearson correlation coefficient (PCC) is used in the update stage of the particle filter (PF) to estimate the moving path of each cluster in order to improve the accuracy in a complex environment with obstacles. The experiments were verified by a SCITOS G5 robot. The results show that this method can achieve an matching rate of 90.18% and a localization accuracy of 0.33m in an environment with the presence of obstacles. This method effectively improves the matching rate and localization accuracy of multiple objects in indoor scenes when compared to the Bray-Curtis (BC) similarity matching-based approach as well as the particle filter-based approach.

## 1. Introduction

In recent years, with the development of wireless communication technology, location-based services have been widely used in search and rescue, medical services, intelligent transportation, logistics management, and other fields [1], and many positioning technologies have been investigated in the research community. Among them, it is very important to identify and localize the dynamic object in the indoor environment. This field has a wide range of applications, such as monitoring teams with different identities, and tracking designated objects, library book management, real-time control of equipment and participants in the venue, etc. [2].

Identification and localization are usually regarded as two separate tasks, which are solved by different methods. In this field, it is common to deploy cameras in the environment in order to identify and locate objects. Xu et al. [3] realized the positioning of indoor mobile robots by installing the camera above the robot head and extracting the ceiling features. Wang et al. [4] constructed a three-diensional (3D) human tracking system by fusing the information of visual and ultrasonic sensors. Liu et al. [5] analyzed target tracking under different illumination conditions in intelligent monitoring of public places. Although the vision-based method can obtain better positioning accuracy and recognition rate, however, it usually requires clear images to identify and localize, this kind of method has great limitations in practical application, because it must overcome occlusion, motion uncertainty, and environmental appearance changes, and, in some cases, this method may lead to privacy violations. Mostly, the laser range finder is used in the field of object localization due to its high precision, wide range, and fast transmission speed. However, it can only obtain sparse environmental information, and it is difficult to distinguish objects with similar appearance. It is necessary to extract effective features from the sparse information for identification, which makes the algorithm very complicated. Radio Frequency Identification (RFID) technology has a unique tag ID, which can quickly identify the object by relying on the radio frequency signal, so it can save a lot of computing resources, and can solve the problems of occlusion and other environmental factors. However, due to its hardware limitation, accurate localization cannot be achieved.

It is difficult to accurately identify and locate the object by only relying on a certain kind of sensor in the indoor environments, for the proper identification and localization it requires the fusion of multiple sensors information. Many researchers have conducted extensive research in this field. Xing et al. [6] designed a multi-sensor information fusion method based on a visual marker called ArUco. This method uses Kalman filter to fuse the information of visual marker, ultrasonic, and inertial sensors to localize micro air vehicles in indoor environments with an accuracy up to 4 cm. You et al. [7] used Unscented Kalman filter to fuse UWB and IMU data for the localization of quadrotor UAV in indoor environments. Li et al. [8] employed sliding window filter (SWF) to fuse camera and IMU data for accurate 3D motion tracking and reconstruction. Peng et al. [9] proposed a multi-sliding window classification adaptive unscented Kalman filter (MWCAUKF) method with timestamp sort updating, which could fuse multiple kinds of sensors data. Shi et al. [10] realized the positioning and navigation of the mobile robot by integrating laser and geomagnetic sensors. Zhao et al. [11] proposed a method that fuses the information obtained by 3D LiDAR and camera. The average identification accuracies of their method for cars and pedestrians are 89.04% and 78.18%, respectively. Digiampaolo et al. [12] proposed a localization system based on the passive signal phase, and used extended Kalman filter (EKF) to fuse RFID and odometer information to achieve the object localization, with an accuracy of up to 4 cm. Tian et al. [13] proposed a low-cost INS and UWB integrated pedestrian tracking system, which only uses single UWB anchor node at an unknown location, minimizing infrastructure cost and setup.

Each frame of data measured by a laser range finder can be used to represent a static environment. Therefore, if each frame of data is correlated based on time series, the localization of dynamic objects in the environment can be achieved [14,15]. Tang et al. [16] designed a real-time indoor positioning system based on laser scan matching for unmanned ground vehicles in a large area. Zhang et al. [17] designed an object localization algorithm based on laser range finder, which realized the object localization under the airborne reconnaissance platform. However, when we use the laser range finder to identify the object, as mentioned above, it is difficult to distinguish objects with similar appearance, so there is a certain degree of singularity [18]. Researchers have done a lot of research in using laser measurement for object identification. Wang et al. [19] used 3D laser sensors and sliding windows to achieve object detection and identification. Huang et al. [20] proposed using feature fusion and establishing spatio-temporal feature vectors to realize the detection and recognition of dynamic obstacles, with a recognition rate of up to 87.7%. Tong et al. [21] proposed an object recognition method by combining laser and infrared information. This method can achieve more than 90% recognition accuracy for objects with large shape differences, but the recognition accuracy for objects with similar shapes is low.

RFID is widely used in the field of object identification due to its characteristics of fast recognition speed, high recognition accuracy, and wide coverage. Liu et al. [22] proposed a method for quickly tracking dynamic targets based on the RSS measurements from a pair of RFID antennas. Fan et al. [23] proposed a method, called “$M^{2}AI$”, which uses convolutional neural networks for deep learning to achieve multiple objects identification. The identification rate of this method can reach 97%, but, as the number of dynamic objects (for example three objects) increases, the recognition rate will drop to about 80%.

The unique ID of the RFID tag can be used to solve the singularity problem of laser sensor in object identification. The advantages of high measurement accuracy of laser range finder can be used to make up for the low positioning accuracy of RFID. Therefore, we fuse information from RFID and laser range finder to achieve dynamic multiple objects identification and localization. More specifically, we use the phase difference of RFID in the adjacent time to calculate the radial velocities of moving tags. Meanwhile, after clustering the laser points, we use the Pearson correlation coefficient in the update stage of particle filter to estimate the moving path and radial velocity of each cluster. Through the similarity matching algorithm based on the sliding time window, we can match the laser cluster-based radial velocities and the phase-based radial velocities in each window, and find the cluster with the highest similarity for each dynamic RFID tag, and the center coordinate of the cluster is considered as the position of the object.

We summarize the contributions of this article, as follows.

(1) We present a solution that incorporates RFID phase information and laser range measurements for multiple dynamic objects identification and localization in indoor environments.
(2) We propose incorporating the Pearson correlation coefficient into the update stage of particle filtering. This method can effectively estimate the historical trajectories of moving objects in an environment with obstacles.
(3) We set up different paths in different environments on campus, and thoroughly evaluated our method. Our method can effectively identify and locate the passing pedestrians in indoor scenes when compared to the Bray–Curtis (BC) similarity matching-based approach as well as the particle filter-based approach.

We organize the subsequent sections of this paper, as follows. The related work is discussed in Section 2. An overview of the system is described in Section 3. In Section 4, we present the details of the dynamic multi-object identification and localization method. We show the experimental results in Section 5 and conclude the paper with possible extensions in Section 6.

## 2. Related Work

This section gives a thorough overview of the work related to the identification and localization of dynamic objects using RFID techniques.

Dynamic objects localization approach is widely used in intelligent monitoring, human-robot interaction, virtual reality, and robot navigation. Typical methods for localizing dynamic objects using RFID systems are the LANDMARK-based approach [24], the SpotON-based approach [25], and the VIRE(VIrtual Reference Elimination)-based approach [26]. When the LANDMARC system is used for localization, there is uncertainty in the selection of the number *K* of neighbor labels. Besides, it is necessary to add additional reference tags in order to obtain a more accurate localization result, but too many tags will cause signal interference between tags, which will affect the positioning accuracy of the system. The SpotON method measures the RSS of a series of tags, and establishes the regression equation of the distance between RSS and the tag to the reader antenna, and then estimates the distance from the target to the antenna through RSS, and finally determines the location of the target by trigonometry using the ranging information of multiple antennas. However, RSS distance model needs to be built in advance, which is a heavy workload. The VIRE algorithm uses linear interpolation to replace the virtual position with the real position to improve the system accuracy. However, the real RSS value changes non-linearly. There is an error between the virtual position and the real position while using linear mathematical interpolation. In addition, the VIRE algorithm does not have a good localization effect on the border area. The calculation cost will be increased if additional real labels are added to the border area.

Many researchers combine RFID with other sensors to realize the identification and localization of dynamic objects, as it is difficult to meet the requirements of high positioning accuracy and low cost by only using RFID system. Alvarado et al. [27] combined a RFID system, an omnidirectional Mobotix C25 camera, and a laser range finder to realize the localization of tour-guide robot. Choi et al. [28] combined RFID system and ultrasonic sensors to overcome uncertainties in previous RFID systems for mobile robot localization. Suparyanto et al. [29] presented a system to localize container truck while using indoor Global Positioning System (iGPS), RFID, and Inertial Measurement Unit (IMU) consisting of accelerometer, gyroscope, and magnetometer. Wang et al. [30] and Li et al. [31] combined the RFID and Kinect camera to realize the identification and localization of multiple dynamic objects, but its accuracy is low. Parr et al. [32] realized tag tracking by combining the information of RFID and IMU. Faramondi et al. [33] combined the information of IMU, RFID, and triad-magnetometer in order to realize the identification and localization of rescuers, but the accuracy is low. In this paper, we show that we can achieve the identification and localization of multiple dynamic objects with high accuracy by fusing the information from RFID and laser range finder.

## 3. System Overview

**Figure 1:**
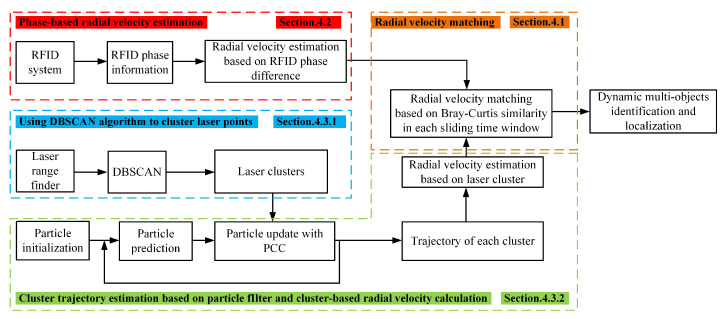
System overview.

If an object is attached with an RFID tag, the identification of this object is known to us. However, we cannot know the exact location of the object in an environment due to the physical limitations of the RFID system. In other words, we only know that there are several objects in the environment, but we can’t distinguish them. Therefore, this paper proposes a method for localizing multiple dynamic objects with known identities in an indoor environment by matching laser clusters with RFID tag IDs. The entire system uses RFID and two-dimensinal (2D) laser range finder to measure obstacles and moving objects in the environment, as shown in Figure 1. Subsequently, the algorithm uses the phases collected from the RFID to calculate the phase-based radial velocity of each RFID tag, and uses laser range finder data to calculate the laser cluster-based radial velocity of each cluster. In addition, we use Pearson correlation coefficient combined with particle filter to realize the tracking of a cluster in each sliding time window. Finally, the radial velocity similarity matching is used to locate the dynamic targets with known identities.

More specifically, we collect the measurements through RFID reader and 2D laser range finder. The RFID reader controls the antenna to scan the tagged dynamic objects in the environment, and the 2D laser range finder is used to measure the distance and angle from the surrounding obstacles in an environment. Afterwards, the information that is detected by the two sensors is stored the database. We calculate the moving object’s radial velocity based on the phase information reported from the RFID reader; meanwhile, we use the DBSCAN algorithm to cluster the laser ranging information. After obtaining the cluster information, we use the Pearson correlation coefficient and particle filter to estimate the moving path of each cluster, and estimate the radial velocities of clusters based on the distance between the two clusters at adjacent moments. Then, we define a fixed-size sliding time window *w* with the current time *T* as the end, and use the Bray–Curtis similarity algorithm to constantly matching the radial velocity estimated by two sensors in each window. According to the matching results, we select the cluster with highest similarity to realize the identification and localization of multiple dynamic objects. The specific implementation method will be described in detail in the next part of this paper.

## 4. Indoor Multiple Dynamic Objects Identification and Localization

### 4.1. Object Identification and Localization Based on Radial Velocity Matching

Restricted by the RFID physical system, when we estimate the information between the object and RFID antenna, we can only obtain the phase information, and it is difficult to estimate the exact coordinates of the object based on phase. However, we can infer the radial velocity of an object based on the phase difference. Radial velocity refers to the velocity component of the object’s movement velocity in the direction of the observer’s line of sight, which is, the projection of the velocity vector in the direction of the line of sight, which is often used to represent the rate of change of the distance from the object to the observation point. For the same observation point, assuming that there are multiple objects in the environment, the objects cannot be in the same position at the same time, which means that their distance and angle to the observation point are different, resulting in their radial velocity being different from each other. Therefore, we use laser sensor to estimate the radial velocity of each cluster in the environment in order to better match the object’s radial velocity estimated by the RFID system. The similarity of two velocities are compared to realize the identification and localization of the moving objects. Different applications often need to use different similarity measurement methods.

Different similarity measures are often used in different applications. In this paper, it is necessary to find a reliable similarity measurement function to match the speed estimated by RFID due to the large number of clusters obtained by laser sensors. Vorst et al. [34,35] compared different similarity algorithms in order to achieve self-localization with passive RFID fingerprints. They showed that the Bray–Curtis (BC) measure gives the best performance among all of the evaluated methods. The Bray–Curtis similarity algorithm [36] is often used to calculate the similarity between different samples. Samantaray et al. [37] used BC similarity to effectively realize medical image recognition and retrieval. Therefore, we use BC similarity to match the radial velocity. Besides, in order to achieve better results, this article refers to the sliding window method commonly used in image object identification. By using sliding time windows to limit the number of laser frames, we ensure that all of the objects can be identified in each window. This paper defines a fixed-size sliding time window *w* with the current time *T* as the end, constantly matching the radial velocity estimated by two sensors in each window. The window range is denoted as [*T-w+1*:*T*]. The similarity of laser cluster-based radial velocity and RFID phase-based radial velocity can be computed as:

(1) $$Sim=(1+\frac{1}{w}\sum_{t=T}^{T-w+1}\frac{|V_{t}^{R}-V_{t}^{L,i}|}{|V_{t}^{R}\left|+\right|V_{t}^{L,i}|}),$$

where *i* represents the *i*-th cluster at the current time, and $V_{t}^{L,i}$ represents its radial velocity at time *t*. $V_{t}^{R}$ represents the phase-based radial velocity at time *t*. In addition, if the radial velocities that are estimated by laser and RFID belong to the same object, the similarity should be high. Therefore, we choose the cluster which has the highest similarity and assigns the tag ID to it. The central coordinate of this cluster is considered as the estimated position of the moving object, thereby realizing the identification and localization of objects.

Estimating the radial velocities of moving tags based on RFID phase, and radial velocities estimation method based on laser clusters will be given in Section 4.2 and Section 4.3, respectively. Table 1 lists the symbols in this paper and their meanings.

### 4.2. Estimating the Radial Velocities of Moving Tags Based on RFID Phase Difference

This paper uses the RFID phase information to estimate the radial velocities of the moving tags. The RFID phase information is a periodic function of the distance between the tag and the antenna (the period is 2π) and is given by:

(2) $$\theta_{t}=2\pi \cdot \frac{2\cdot d_{t}}{\lambda }\cdot mod\left(2\pi \right),$$

where $\theta_{t}$ is the signal phase at time *t*, λ is the wavelength of the receiving signal, and $d_{t}$ is the radial distance from the moving tag to the antenna at time *t*. In this paper, the phase differences at adjacent times are first processed and normalized to the main value interval of [−π,π]. The specific implementation is given by:

(3) $$\Delta \theta^{{}^{\prime }}=\left\{\begin{array}{cc} \Delta \theta , & -\pi <\Delta \theta <\pi \\ \Delta \theta -2\pi , & \Delta \theta \geq \pi \\ \Delta \theta +2\pi , & \Delta \theta \leq -\pi , \end{array}\right.$$

We may get two different values with a phase difference of π when a tag is not moving [38] due to the limitation of the RFID signal processing algorithm. Therefore, it is necessary to set an appropriate threshold to eliminate the effect of the π phase jump. This paper assumes that the phase difference of the same object should not exceed the threshold φ in the adjacent time. When the phase difference is between −φ to φ, we use this phase difference to estimate the radial velocity, otherwise we set the radial velocity to an invalid value. The radial velocity of each tag estimated by the RFID at the current moment can be computed as:

(4) $$V_{t}^{R}=\frac{\Delta \theta^{{}^{\prime }}}{4\pi \Delta t}\cdot \lambda ,$$

### 4.3. Estimiating the Radial Velocities of Laser Clusters

#### 4.3.1. Laser Clustering Based on DBSCAN

**Figure 2:**
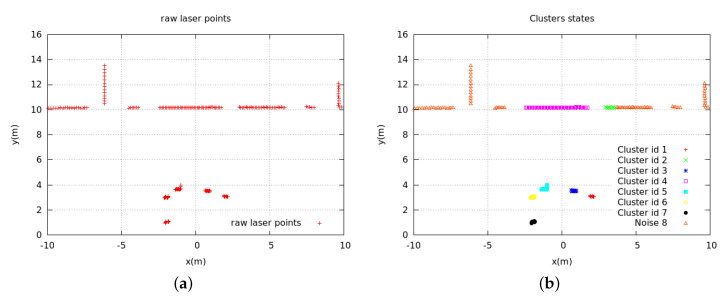
Clustering results. (**a**) raw laser points; (**b**) DBSCAN results.

The DBSCAN algorithm is a spatial clustering algorithm based on density [39]. The remarkable advantage of this algorithm is that the algorithm is fast, and it can divide the regions with enough high density into a group, which effectively deals with noise points and quickly finds spatial clustering of arbitrary shape. The experiment divides the laser points into core points, boundary points, and noise points. For a given point, if the number of adjacent points in the neighborhood with radius ρ is greater than ξ, then this point is regarded as the core point. If it is within the ρ neighborhood of the core point, we treat it as a boundary point; otherwise, we treat it as a noise point. After clarifying the categories of the respective laser points, the core points and the boundary points are merged into one cluster. Finally, we will get $n_{t}$ clusters, where $C_{t}=C_{t}^{1},C_{t}^{2},\ldots ,C_{t}^{\left(n_{t}\right)}$, and we can estimate the central coordinate of each cluster as: ${\bar{P}}_{t}^{\left(j\right)}=\left({\bar{x}}_{t}^{\left(j\right)},{\bar{y}}_{t}^{\left(j\right)}\right),j\in \left[1:n_{t}\right]$. Figure 2 shows one example of the clustering result.

The two most important parameters in DBSCAN algorithm are the radius ρ and number of laser spots ξ in each cluster. Our previously published paper [40] showed a comparison of the clustering performance under different ρ and ξ. The parameters of DBSCAN are not discussed in the article in order to save the space of the paper. According to past experience, we set the parameters of DBSCAN as ρ = 0.1, ξ = 2.

#### 4.3.2. Cluster Trajectory Estimation Based on Particle Filter

We need to calculate the radial velocity of each cluster in order to match the radial velocity estimated by RFID. Therefore, it is necessary to find the historical trajectory of each cluster at the previous moment. The particle filter is widely used in the field of navigation and positioning due to its advantages of high robustness, high accuracy, and excellent performance in non-linear and non-Gaussian systems. In this paper, we set up an independent particle filter to track the trajectory of each cluster. In each particle filter, the position of a moving object can be represented by a set of weighted particles: $X_{i,t}={\left\{X_{i,t}^{\left[n\right]},w_{i,t}^{\left[n\right]}\right\}}_{n=1}^{N}$, where *N* represents the number of particles, *i* represents the particle filter of the *i*-th object, and $i\in \left[1:n_{t}\right]$. Besides, $X_{i,t}^{\left[n\right]}=x_{i,t}^{\left[n\right]},y_{i,t}^{\left[n\right]}$ represents the two-dimensional (2D) coordinates of each particle, and $w_{i,t}^{\left[n\right]}$ represents its weight. The particle filter will predict and update iteratively based on measurements arrival.

- Prediction

In this paper, we use the position $X_{i,t}^{\left[n\right]}$ of the particles at time *t* and the motion model to estimate the position $X_{i,t-1}^{\left[n\right]}$ in the previous time. The Gaussian function is chosen as the model of motion prediction and the corresponding parameters of the Gaussian function are used to adjust the particle distribution density because of the uncertainty of the direction and velocity of the moving object. The prediction is performed with the following:

(5) $$\left\{\begin{array}{c} x_{i,t-1}^{\left[n\right]}=x_{i,t}^{\left[n\right]}+N\left(0,\sigma \right)\\ y_{i,t-1}^{\left[n\right]}=y_{i,t}^{\left[n\right]}+N\left(0,\sigma \right), \end{array}\right.$$

where N(0,σ) is the Gaussian noise with zero mean and standard deviation of σ. Because the sampling time interval of the laser range finder is 0.1 s, we assume that the maximum moving distance of an object in each sampling time will not exceed 0.1 m, so we let σ = 0.1. After the prediction, we use the Pearson correlation coefficient in the update stage to match the motion model in Equation (5), to adjust the weight of the particles, thereby correcting the prediction information.

- Update

The update stage of particle filter is to use the measurement value of every moment in historical time to update the particle’s weight. In the prediction stage, we generate particles with Gaussian distribution, so, in the update stage, we use the probability density function of Gaussian distribution to update the particle’s weight. We calculated the distance between the cluster and the *n*-th particle. Therefore, the weight $w_{i,t-1}^{\left[n\right]}$ of each particle in ordinary particle filter is updated according to the following:

(6) $$w_{i,t-1}^{\left[n\right]}=\mu \cdot w_{i,t}^{\left[n\right]}\cdot \frac{1}{n_{t-1}}\sum_{i=1}^{n_{t-1}}\frac{1}{\sqrt{2\pi }\sigma }\cdot \exp\left(-\frac{\Delta d^{2}}{2\tau^{2}}\right),$$

where

(7) $$\Delta d=\sqrt{{\left(x_{i,t}^{\left[n\right]}-{\bar{x}}_{t-1}^{\left(j\right)}\right)}^{2}+{\left(y_{i,t}^{\left[n\right]}-{\bar{y}}_{t-1}^{\left(j\right)}\right)}^{2}},$$

where $n_{t}$ indicates that there are $n_{t}$ clusters in the time *t*, Δd represents the distance between the cluster and the particle, τ is the translational coefficient of the distance from particle to cluster, and μ is the normalization coefficient. In this paper, we assume that the radius of a human cluster is 0.1 m, therefore, we set τ = 0.1. The estimated position of each object at time *t* can be obtained by the weighted average of all particles.

Traditional particle filter generally uses a single feature to construct object’s model, but such a model often has different disadvantages due to different features selected: for example, using color features, when the color of the object is similar to the background, the tracking process will become unstable; using the contour or texture features, if the object rotates, deforms or occludes in the process of moving, the tracking effect will become poor or even the tracking fails. This is because, in all of the above cases, when the traditional particle filter algorithm processes the weight of particles in the update stage, it cannot correctly reflect the degree of matching between the particles and the objects. In the resampling stage, after many iterations, there are too few effective particles to approximate the real state of the object, which makes the particles easily drift to the obstacles, leading to tracking failure. To solve this problem, we introduce the Pearson correlation coefficient [41]. It is usually used for the indoor localization by comparing the similarity between the location fingerprint and a known fingerprint database [42]. In addition, it is often used in the template matching stage of image processing in order to compare the similarity between the two images [43]. In this paper, we compare the *i*-th cluster at time *t* with the clusters in each historical time to obtain the Pearson correlation coefficient. Afterwards, we substitute the results into the probability density function. The updated weight of the particle is computed as:

(8) $$w_{i,t-1}^{\left[n\right]}=\mu \cdot w_{i,t}^{\left[n\right]}\cdot \frac{1}{n_{t-1}}\sum_{i=1}^{n_{t-1}}\left(1-p\right)\cdot \exp\left(-\frac{\Delta d^{2}}{2\tau^{2}}\right),$$

where

(9) $$p=\frac{\sum_{n=1}^{N}\left(x_{i,t}^{\left[n\right]}-{\bar{x}}_{t-1}^{\left(j\right)}\right)\cdot \left(y_{i,t}^{\left[n\right]}-{\bar{y}}_{t-1}^{\left(j\right)}\right)}{\sqrt{\sum_{n=1}^{N}{\left(x_{i,t}^{\left[n\right]}-{\bar{x}}_{t-1}^{\left(j\right)}\right)}^{2}}\cdot \sqrt{\sum_{n=1}^{N}{\left(y_{i,t}^{\left[n\right]}-{\bar{y}}_{t-1}^{\left(j\right)}\right)}^{2}}},$$

where $\left\{{\bar{x}}_{t-1}^{\left(j\right)},{\bar{y}}_{t-1}^{\left(j\right)}\right\}$ represents the center coordinate of the *j*-th cluster at the historical time, and $j\in [i:n_{t}]$.

Lastly, the resampling tackles the issue of particle degradation that occurs after many iterations. We replicate a series of particle sets by eliminating particles with small weights and replicating particles with large weights.

#### 4.3.3. Calculating Each Cluster’s Radial Velocity

Now we have each cluster’s historical position, then we need to calculate the clusters of the same cluster moving in the adjacent time, the radial velocity of cluster at each time can be expressed as:

(10) $$V_{t}^{\left(i\right)}=\frac{\sqrt{{\left({\bar{x}}_{t}^{\left(i\right)}\right)}^{2}+{\left({\bar{y}}_{t}^{\left(i\right)}\right)}^{2}}-\sqrt{{\left({\bar{x}}_{t-1}^{\left(i^{{}^{\prime }}\right)}\right)}^{2}+{\left({\bar{y}}_{t-1}^{\left(i^{{}^{\prime }}\right)}\right)}^{2}}}{\Delta t},$$

where $\Delta_{t}$ represents the time difference between adjacent times.

In addition, the experimental results obtained by the other two methods (namely BC-based method and PF-based method) are compared. In the first method, we only use the BC similarity algorithm to match the radial velocity estimated by two sensors in each sliding time window. Different from the algorithm proposed in this paper, the BC-based method does not use particle filter to estimate the historical information of cluster, but it finds the nearest cluster $C_{t-1}^{\left(i^{{}^{\prime }}\right)}$ in the previous time t−1 for each cluster $C_{t}^{\left(i\right)}$ of current time *t*. In the BC-based method, we assume that the moving distance of the same object should be very small at the adjacent time. Therefore, the previous cluster of a moving object (denoted as $i^{{}^{\prime }}$ ) can be determined by:

(11) $$i^{{}^{\prime }}=arg\min_{j}\sqrt{{\left({\bar{x}}_{t}^{\left(i\right)}-{\bar{x}}_{t-1}^{\left(j\right)}\right)}^{2}+{\left({\bar{y}}_{t}^{\left(i\right)}-{\bar{y}}_{t-1}^{\left(j\right)}\right)}^{2}},$$

After finding the historical information of each cluster, the radial velocity of each cluster can be calculated by Equation (10), and matched with the object’s phased-based radial velocity by Equation (1).

As for the second method, we use the traditional particle filter described above, and use the traditional method to update the weight of particles (Equation (6)). Similarly, after obtaining the historical information of each cluster, we also calculate the radial velocity of the cluster by Equation (10), and it performs similarity matching by Equation (1).

## 5. Experimental Results Analysis

### 5.1. Experimental Setups

**Figure 3:**
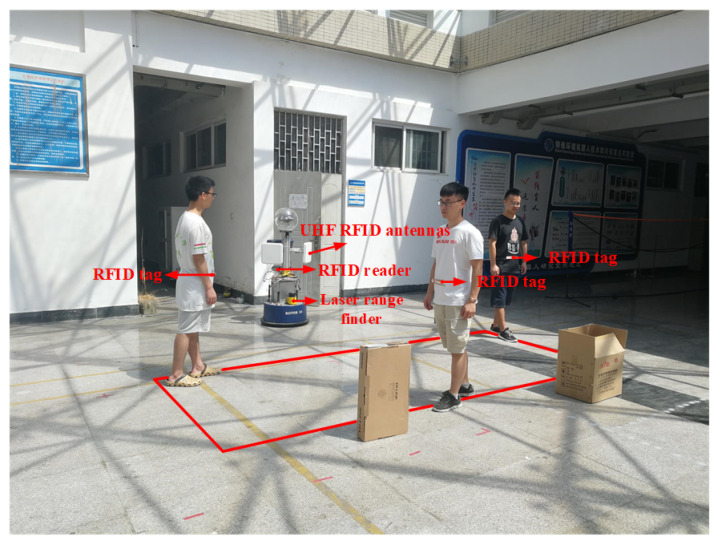
Experimental setup.

The feasibility of the proposed method in this paper is verified by using the SCITOS G5 robot. We conducted experiments in an indoor environment that is shown in Figure 3. This robot integrates a UHF RFID reader, named Impinj Speedway Revolution R420, two circularly polarized antennas of the type Laird Technologies S9028, and a laser range finder, named SICK S300. The RFID reader provides a maximum measuring distance up to 10 m. The laser range finder provides a maximum measuring distance up to 29 m and a horizontal scanning angle of $270^{\circ }$ with a scanning resolution of $0.5^{\circ }$.

The experiments have been tested in a space that is a rectangular area of 4 m × 2 m, as shown in Figure 3. The robot had been placed one-meter perpendicular to the rectangle’s long side. During the experiment, the position of the robot remained unchanged. The RFID tags worn by the three persons moved along the edge of the rectangle. Each of them walked three circles, and each person’s speed was different (but each person’s speed was kept constant while walking). We set a marker every one meter on the ground shown by the red line in the Figure 3 in order to calculate the positioning error. Each time the experimenter went across to a mark, the software records the time when he arrives and stores it in the database. We calculate the average velocity of the experimenter between the two adjacent markers, calculate the coordinates of their position at each moment, and treat this coordinate as the true position of the experimenter. Because the distance between adjacent marks is short, the ground truth error can be ignored. Subsequently, the position estimated by the algorithm is compared with the ground truth to measure the localization error, which is defined as the Euclidean distance between the estimated position and the true position. We think that, if the error is less than 0.8m, the cluster and tag ID are successfully matched. The matching rate is obtained by comparing the number of clusters successfully matched with the total number of clusters.

### 5.2. Impact of Different Methods on Experimental Results

**Figure 4:**
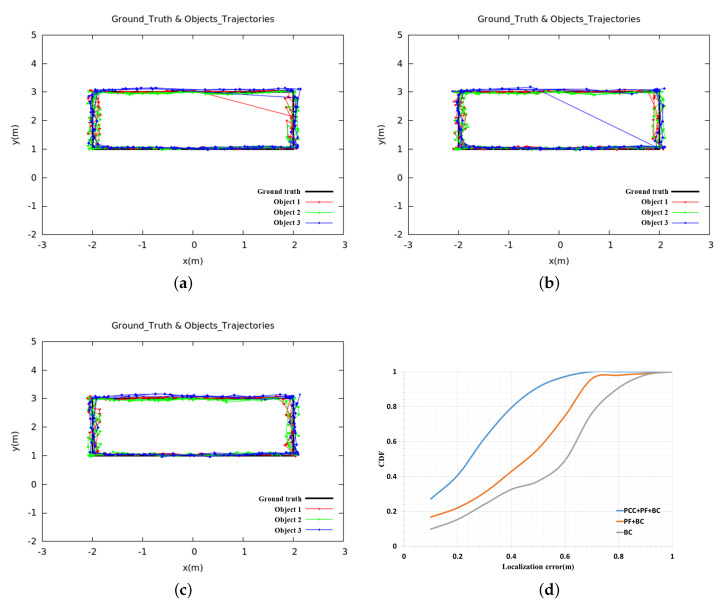
Comparision of the estimated trajectories based on different approaches. (**a**) BC; (**b**) PF + BC; (**c**) PCC + PF + BC; (**d**) CDFs.

In this section, we used three different methods to compare the final localization accuracy and matching rate. In the first method (BC), we only use Bray–Curtis similarity to match the radial velocities estimated by two sensors, respectively. For the second method (PF + BC), we only use particle filter to estimate the historical coordinates of each cluster. As for the third method (PCC + PF + BC), we added the Pearson correlation coefficient in the update stage of particle filter to further constrain the particles. We set ρ = 0.1, ξ = 2, *w* = 25, *N* = 200, $\varphi =90^{\circ }$, and the results of the three methods are compared in Table 2, and the estimated trajectories of moving objects are shown in Figure 4.

If the obstacles are present in the certain environment, the BC-based approach cannot locate the moving objects very well, as shown in Table 2 and Figure 4. The localization error is 0.76 m, and the identification rate is 79.2%. From Figure 4a, it can be seen that the object’s trajectory jumps greatly. That is because we assume that the moving distance of the same object at adjacent times should be the smallest, so we find the cluster $C_{t-1}^{\left(i^{{}^{\prime }}\right)}$ with minimal distance at the previous time t−1 for each cluster $C_{t}^{\left(i\right)}$ at time *t* through Equation (11). When the distance between the object and the obstacle is very close, the algorithm may incorrectly obtain the historical information of the moving object, which causes the radial velocity matching to fail. Only after the object is far away from the obstacle by a certain distance, the algorithm can recover, trajectory jump of BC-based approach as shown in Figure 4a. Although the particle filter-based approach improves the accuracy and matching rate compared with the BC-based approach, the result is still not good enough: the localization error is 0.65 m and the matching rate is only 83.1%. We use the traditional particle filter method to update the particle’s weight (Equation (6)), and then obtain the historical information of each object. Because particle filter mainly uses laser data to estimate the historical trajectory of the object, if human moves close to or away from the obstacles during the movement, the performance of the system will become very poor. This is because, in this case, the traditional particle filter algorithm cannot correctly reflect the matching degree between the particle and the object when processing the weight of the particle in the update stage. In the resampling stage, after many iterations, there are too few effective particles to approximate the true state of the object, which makes the historical position estimation of the object deviate, which further leads to the failure of radial velocity matching and trajectory jump. From Figure 4b, we can also clearly see the trajectory jump. The method of combining PCC and particle filter can better constrain the particles, the localization error is reduced from 0.65 m to 0.33 m, and the matching rate is increased from 83.1% to 90.2%. When compared with Figure 4a,b, the objects trajectories that are estimated by this method (Figure 4c) are smoother. Besides, if an object moves along the circular arc equidistance from the RFID antenna, there will be no difference in distance, and thus the radial velocity tends to zero, which is also one of the sources of error in this research.

### 5.3. Impact of Different Parameters on Experimental Results

#### 5.3.1. The Influence of Antenna Settings on Experimental Results

We evaluated the localization error under various antenna combinations, as the detection range of RFID is directly confined by antennas on robot. We set ρ = 0.1, ξ = 2, *w* = 25, *N* = 200, $\varphi =90^{\circ }$, and the result is shown in Table 3. Due to the limitation of the coverage of the RFID antenna, the localization accuracy based only one antenna is low (for example, when only the right antenna is used, the localization error is 0.82 m). However, when we use two antennas, the measurement range of the antenna can completely cover the entire experimental environment, and the localization error is reduced from 0.82 m to 0.33 m. Therefore, two antennas are used in this paper for the localization and identification of the dynamic multi-objects.

#### 5.3.2. The Influence of Phase Shift Threshold φ on Experimental Results

An appropriate setting of the RFID phase threshold is the key to the algorithm for eliminating π phase jump and accurately estimating the object’s moving radial velocity. In this paper, it is assumed that the phase difference of the same object in the adjacent time should not exceed the threshold. In this section, we set ρ = 0.1, ξ = 2, *w* = 25, *N* = 200. Table 4 shows the localization accuracy under different phase shift thresholds.

It can be seen from Table 4 that when φ is set too small (e.g., $\varphi =10^{\circ }$), the algorithm will erroneously remove the originally correct phase information, resulting in the matching rate of only 81.6% and a localization error of 0.72 m. When φ is set too large (e.g., $\varphi =180^{\circ }$), the algorithm cannot correctly handle the phase where the jump occurs, resulting in the localization error up to 0.83 m and matching rate is only 66.8%. Therefore, only by setting an appropriate phase shift threshold can we better eliminate the effect of phase jumps while retaining normal phase information. We set $\varphi =90^{\circ }$, the matching rate can reach 90.2%, and the localization error is only 0.33 m, in order to obtain better experimental results.

#### 5.3.3. The Influence of The Number of Particles *N* on The Experimental Results

In the traditional particle filter algorithm, the number of particles will have an effect on the results. In this paper, Pearson correlation coefficient is added to constrain the particles in the update stage of particle filter, so we carry out experiments to analyze the impact of the number of particles on the localization error and matching rate in this case. We used CPU with core i5-7300 HQ, 2.50 GHz, and 8 GB ram in the experiment, and set other parameters exactly the same as before. Table 5 lists the results.

It can be seen in the above Table 5, a small *N* (such as *N* = 5) gives an increase in the localization error, since the small number of particles cannot effectively represent the probability density. The positioning accuracy gets improved when increasing the number of particles, similarly, performing filtering with the large number of particles also consumes more time. With a large *N* (such as *N* = 1000), we almost get the same localization results. For considering the accuracy and the time-consuming of the algorithm, we choose *N* = 200 in our experiment.

#### 5.3.4. The Influence of The Time Window Size w

The size of time window directly affects whether all moving objects can be found in each window. In the experiment, we set ρ = 0.1, ξ = 2, $\varphi =90^{\circ }$, *N* = 200, and the experimental results under different *w* sizes are compared in Table 6.

**Figure 5:**
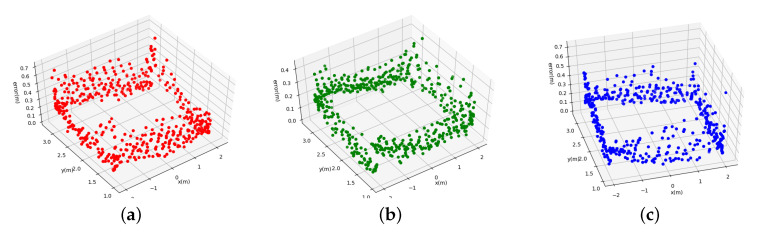
The positioning error at different locations with *w*=25. (**a**) The first object; (**b**) The second object; (**c**) The third object.

It can be seen from Table 6 that, as compared with the other two methods (BC and PF + BC), our proposed method (PCC + PF + BC) integrates Pearson correlation coefficient into the update stage of particle filter, and matches the laser cluster-based radial velocity and the phase-based radial velocity in each window has the least localization error and the highest matching rate. In addition, if the window is too small (e.g., *w* = 5), when the object is occluded during the experiment, the algorithm cannot find all objects in a period time, which will cause the failure of radial velocity matching. Consequently, we obtain the error up to 0.71 m, and the matching rate reduces to 81.8%. A suitable window size (e.g., *w* = 25) can ensure that all moving objects can be found in each time window, the matching rate can be 90.2%, and the localization error is only 0.33 m. If the window is too large (e.g., *w* = 50), the algorithm can always filter out the appropriate cluster to match with RFID tag in each window, which has little impact on the experimental results, but redundant information may occupy the computing resources of the system, and affect the real-time performance of localization and identification. Besides, as compared with *w* = 25, the average time-consuming is increased to 62.24 ms. Therefore, it is very important to choose an appropriate window size. This paper uses the window size *w* = 25 in order to ensure that the experiment has small positioning error and high matching rate. Figure 5a–c shows the localization error at different locations with *w* = 25.

### 5.4. Evaluation of the Approach with a Complex Path and a Different Environment

**Figure 6:**
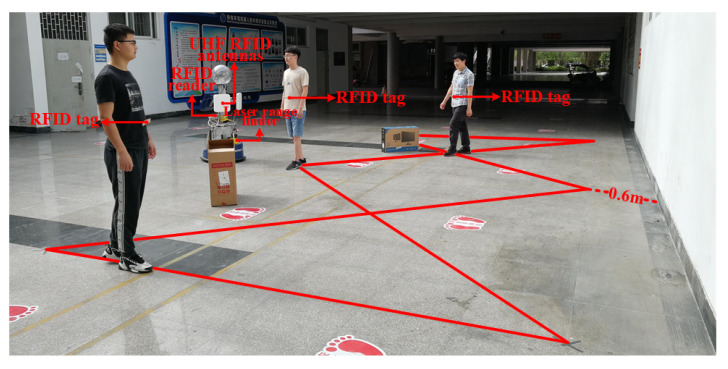
Complex paths experimental setup.

**Figure 7:**
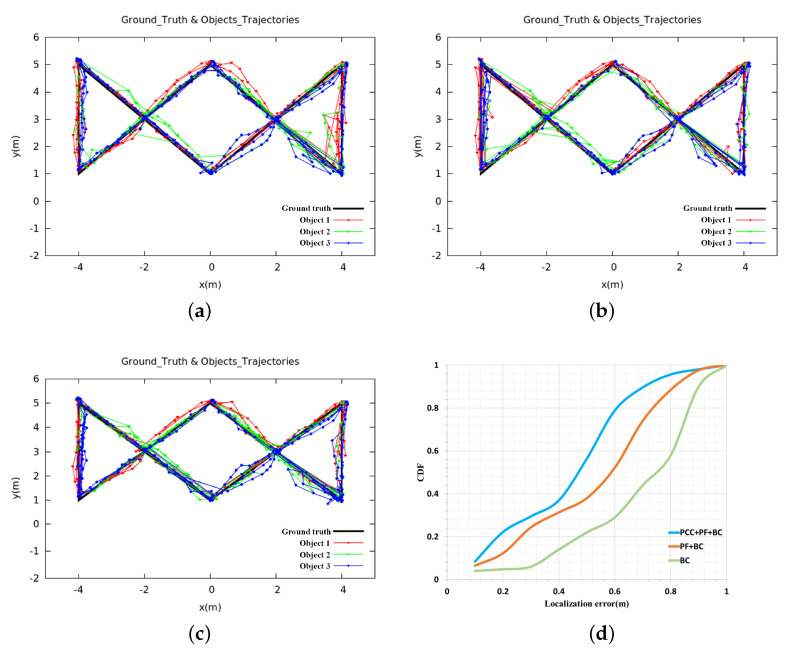
Complex paths results. (**a**) BC; (**b**) PF + BC; (**c**) PCC + PF + BC; (**d**) CDFs.

We expand the original experimental scene to an area of 8 m × 4 m and move it to the area closer to the wall in order to verify the robustness of the whole system. The specific experimental scenario is shown in Figure 6. The experimental parameters are set to be the same as the original experimental parameters, and the result is shown in Figure 7. As can be seen from Figure 7c, as compared with Figure 7a,b, the object’s estimated paths are basically consistent to the ground truth, and the average localization error of all humans is 0.52 m, which is only 0.19 m worse than the original experiment. The BC-based approach (BC) cannot locate the moving objects very well, the localization error is 0.90 m. Although the particle filter-based approach (PF + BC) improves the accuracy when compared to the BC-based approach, the result is still not good enough: the localization error is 0.76 m. The experimental results show that our approach is able to achieve identification and localization of multiple objects with good positioning accuracy.

**Figure 8:**
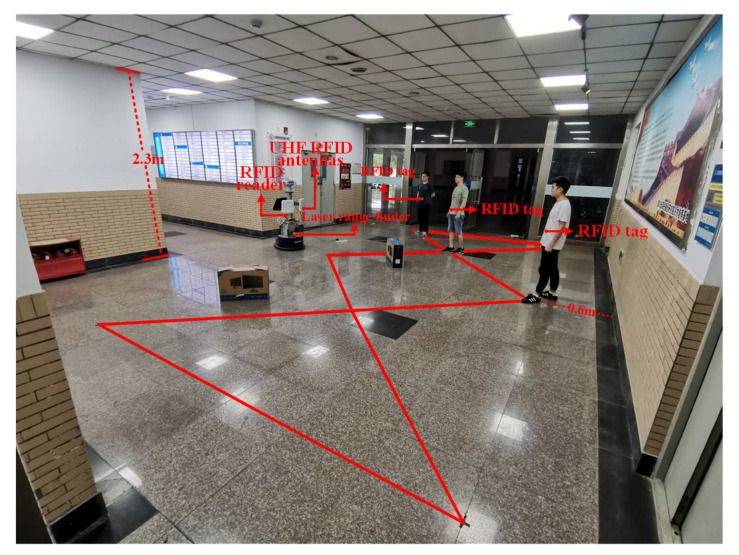
Experimental setup of complex path in indoor environment.

**Figure 9:**
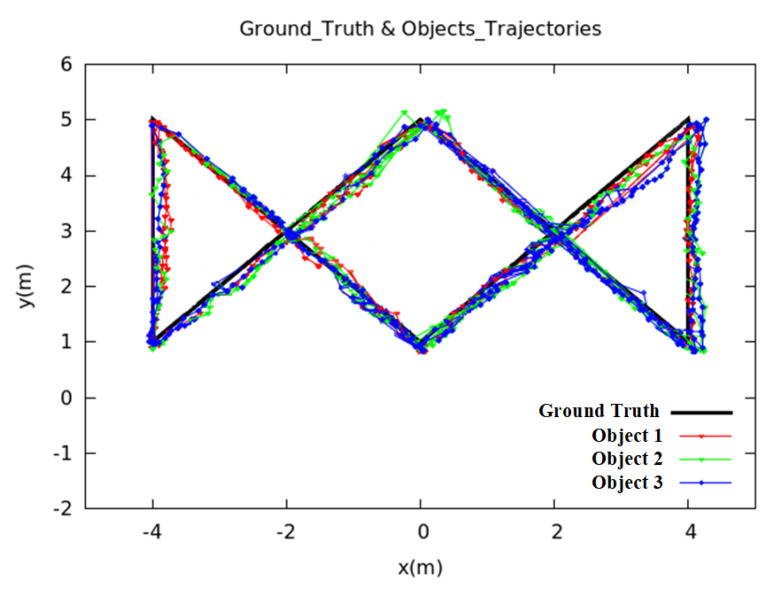
Experimental results of complex path in indoor environment.

We conducted experiments in the lobby of our campus building in order to verify the actual use of our system in an indoor environment. In this experiment scene, the ceiling is about 2.3 m above the ground, and there are several walls in the environment. The experimental scenario is shown in Figure 8, the results are shown in Figure 9 and Table 7. As can be seen from Figure 9, the object’s paths estimated by our method (PCC + PF + BC) are basically consistent to the ground truth, and the average localization error is 0.44 m, which is similar to our previous experiments. Similarly, we also compare the localization error of the other two methods (the BC-based approach (BC), and the particle filter-based approach (PF + BC)), as shown in Table 7.

The experimental results show that our approach is able to achieve identification and localization of multiple objects with a similar localization accuracy when compared to our previous experiments. In practical applications, we can deploy this system in such an environment to identify and locate the passing pedestrians.

## 6. Conclusions

This paper proposed an approach for fusing the RFID and laser data in order to achieve dynamic multi-objects identification and localization by combining Pearson correlation coefficients and particle filter. The Pearson correlation coefficient and particle filter are combined to find out the historical path of each cluster, and then the radial velocity is estimated based on the cluster’s position at adjacent times. At the same time, the radial velocity of the moving object is estimated using the phase difference between the adjacent moments of RFID, and those two are matched by the similarity algorithm based on the sliding time window to realize the identification and localization of multiple dynamic objects. The experiments show that the method that is proposed in this paper can achieve a matching rate of 90.2% in an environment with obstacles and a localization error of 0.33 m. In the future, we will overcome the problem of phase ambiguity, and further improve the matching rate and localization accuracy. Another research direction is visual sensors to overcome the problem of positioning failure of moving objects after long-term occlusion.

## Figures and Tables

**Table 1:** Symbols and their meanings.

Mathematical Symbol	Meaning
Sim	The Bray−Curtis similarity between phase-based radial velocity and laser cluster-based radial velocity
*w*	The size of each time window
$V_{t}^{R}$	RFID phase-based radial velocity at time *t*
$V_{t}^{L,i}$	Radial velocity of the *i*-th cluster at time *t*
$\theta_{t}$	The phase of RFID signal at time *t*
$d_{t}$	The radial distance from the moving tag to the antenna at time *t*
Δθ	Phase difference
$\Delta \theta^{{}^{\prime }}$	The phase differences after normalized to the main value interval of [−π:π]
$C_{t}^{n_{t}}$	Clusters in total at time *t*
$\left({\bar{x}}_{t}^{\left(j\right)},{\bar{y}}_{t}^{\left(j\right)}\right)$	Center coordinates of the *j*-th cluster at time *t*
$v_{t}^{\left(i\right)}$	The radial velocity of cluster *i* at time *t*
*N*	Number of particles
$\left(x_{i,t}^{\left[n\right]},y_{i,t}^{\left[n\right]}\right)$	The position of particle *n* to track *i*-th cluster at time *t*
$\omega_{i,t}^{\left[n\right]}$	The weight of particle *n* to track *i*-th cluster at time *t*
N(0,σ)	The Gaussian noise with zero mean and standed deviation of σ
μ	The normalization coefficient

**Table 2:** Comparison of experimental results based on different approaches.

Methods	Localization Error (m)	Matching Rate (%)
BC	0.76	79.2
PF + BC	0.65	83.1
PCC + PF + BC	0.33	90.2

**Table 3:** Comparison of localization accuracy under different antenna settings.

Antenna Combination	Methods	Localization Error (m)
	BC	0.88
Only Right Antenna	PF + BC	0.96
	PCC + PF + BC	0.82
	BC	0.68
Only Left Antenna	PF + BC	0.89
	PCC + PF + BC	0.61
	BC	0.76
Both	PF + BC	0.65
	PCC + PF + BC	0.33

**Table 4:** The influence of phase shift threshold φ on experimental results.

φ	Methods	Localization Error (m)	Matching Rate (%)
$10^{\circ }$	BC	2.41	54.9
	PF + BC	1.98	64.0
	PCC + PF + BC	0.72	81.6
$30^{\circ }$	BC	0.88	66.4
	PF + BC	0.72	82.1
	PCC + PF + BC	0.34	88.2
$60^{\circ }$	BC	0.79	68.1
	PF + BC	0.63	85.2
	PCC + PF + BC	0.36	87.6
$90^{\circ }$	BC	0.76	79.2
	PF + BC	0.65	83.1
	PCC + PF + BC	0.33	90.2
$120^{\circ }$	BC	0.76	78.8
	PF + BC	0.68	81.4
	PCC + PF + BC	0.37	84.2
$150^{\circ }$	BC	0.77	78.9
	PF + BC	0.67	81.8
	PCC + PF + BC	0.38	83.8
$180^{\circ }$	BC	0.86	66.7
	PF + BC	0.81	67.1
	PCC + PF + BC	0.83	66.8

**Table 5:** The influence of the number of particles *N* on the experimental results.

Number of Particles *N*	Localization Error (m)	Time Consumption (ms)
5	1.41	2.96
20	0.44	3.99
50	0.37	5.16
100	0.35	5.82
200	0.33	6.44
400	0.32	8.30
1000	0.33	12.17

**Table 6:** The influence of the time window size *w* on the experimental results.

Window Size *w* (s)	Method	The Average Distance Traveled in Each Window (m)	Localization Error (m)	Matching Rate (%)	Time Consumption (ms)
5	BC	0.93	1.14	66.9	28.16
	PF + BC	0.93	0.88	72.5	34.42
	PCC + PF + BC	0.93	0.71	81.8	42.56
15	BC	2.79	0.86	67.2	30.3
	PF + BC	2.79	0.67	82.8	38.36
	PCC + PF + BC	2.79	0.37	87.0	46.42
25	BC	4.65	0.76	79.2	33.28
	PF + BC	4.65	0.65	83.1	43.1
	PCC + PF + BC	4.65	0.33	90.2	51.07
35	BC	6.51	0.81	68.3	34.69
	PF + BC	6.51	0.70	82.3	46.97
	PCC + PF + BC	6.51	0.33	87.7	54.81
50	BC	9.18	0.83	67.6	38.41
	PF + BC	9.18	0.64	83.8	54.69
	PCC + PF + BC	9.18	0.35	87.6	62.24

**Table 7:** Comparison of experimental results of complex path in indoor environment based on different methods.

Methods	Localization Error (m)
BC	0.82
PF + BC	0.68
PCC + PF + BC	0.44

## Abbreviations

The following abbreviations are used in this manuscript:

UHF	Ultra High Frequency
BC	Bray-Curtis
RFID	Radio Frequency Identification
PF	Particle Filter
PCC	Pearson Correlation coefficent
